# Phytomelatonin: Assisting Plants to Survive and Thrive

**DOI:** 10.3390/molecules20047396

**Published:** 2015-04-22

**Authors:** Russel J. Reiter, Dun-Xian Tan, Zhou Zhou, Maria Helena Coelho Cruz, Lorena Fuentes-Broto, Annia Galano

**Affiliations:** 1 Department of Cellular and Structural Biology, University of Texas Health Science Center, San Antonio, TX 78229, USA; E-Mails: tan@uthscsa.edu (D.-X.T.); LunaZhou00@163.com (Z.Z.); 2 Department of Occupational Health, Third Military Medical University, Chongqing 400038, China; 3 Department of Veterinary Medicine, Faculty of Animal Science and Food Engineering, University of Sao Paolo, Pirassununga 13635-900, Brazil; E-Mail: helenacruz@usp.br; 4 Department of Human Anatomy and Histology University of Zaragoza, Zaragoza E-50009, Spain; E-Mail: lfuentes@unizar.es; 5 Departamento de Quimica Universidad Autonoma Metropolitana-Iztapalapa, Mexico CP09340, Mexico; E-Mail: agalano@prodigy.net.mx

**Keywords:** melatonin, plant growth, root growth, crop production, abiotic stress, biotic stress, *Arabidopsis*, rice, apple, transgenic plants

## Abstract

This review summarizes the advances that have been made in terms of the identified functions of melatonin in plants. Melatonin is an endogenously-produced molecule in all plant species that have been investigated. Its concentration in plant organs varies in different tissues, e.g., roots *versus* leaves, and with their developmental stage. As in animals, the pathway of melatonin synthesis in plants utilizes tryptophan as an essential precursor molecule. Melatonin synthesis is inducible in plants when they are exposed to abiotic stresses (extremes of temperature, toxins, increased soil salinity, drought, *etc.*) as well as to biotic stresses (fungal infection). Melatonin aids plants in terms of root growth, leaf morphology, chlorophyll preservation and fruit development. There is also evidence that exogenously-applied melatonin improves seed germination, plant growth and crop yield and its application to plant products post-harvest shows that melatonin advances fruit ripening and may improve food quality. Since melatonin was only discovered in plants two decades ago, there is still a great deal to learn about the functional significance of melatonin in plants. It is the hope of the authors that the current review will serve as a stimulus for scientists to join the endeavor of clarifying the function of this phylogenetically-ancient molecule in plants and particularly in reference to the mechanisms by which melatonin mediates its multiple actions.

## 1. Introduction

The identification of the tryptophan derivative, *N*-acetyl-5-methoxytryptamine, in bovine pineal tissue in the late 1950s was a major discovery [1,2]. The common name given to this molecule was melatonin because of its ability to cause the aggregation of pigment granules (melanin) in the chromatophores of amphibian skin and the fact that during its conversion from tryptophan one of the intermediates is 5-hydroxytryptamine (serotonin). The chemical characterization of melatonin along with discoveries related to its synthesis [3,4,5,6] and evidence of its actions in mammals [7,8,9,10] established the pineal, an organ previously thought to be vestigial by most scientists, as a legitimate member of the endocrine hierarchy in vertebrates, particularly mammals.

Since the pineal complex only exists in the nervous system of vertebrates [11,12,13,14], and melatonin’s described actions were endocrine in nature [15,16], melatonin was referred to initially as a neurohormone and it was assumed to be only produced in the pineal gland. This image began to change, however, when this indoleamine was identified in other organs as well, for example, in the lateral eyes of vertebrates [17,18]. Subsequently, melatonin has been uncovered in numerous organs [19] and, theoretically at least, it may be synthesized in every vertebrate cell [20]. Moreover, research within the last two decades has definitively established a diversity of functions of this ubiquitously-distributed indoleamine [21,22,23,24,25], many of which are not related to its actions as a hormone and some of which seem not to require melatonin to interact with a receptor [26].

The rapidly-developing field related to the measurement of melatonin and its actions in multiple vertebrate organs was accompanied by the identification of melatonin in invertebrates [27,28,29], which are devoid of pineal gland, and in unicellular plant-like protist (alga) [30]. In the photosynthetic dinoflagellate, *Gonyanlax polyedra* (now renamed *Lingulodinium polyedrum*), which is responsible for the well-known “red tides”, melatonin levels exhibit a circadian rhythm that persists when these organisms are maintained under constant darkness [30]. The presence of melatonin in transitional species such as protists may have prompted the examination of melatonin in plants, although this is unknown because the original descriptions lack such details since the findings are only published in abstract form [31].

The first comprehensive reports of melatonin in the organs of land plants were published virtually simultaneously by two separate groups in 1995. Dubbels and colleagues [32] undertook their investigation since serotonin, which had been identified in fruits several decades earlier [33,34], is an essential intermediate in the synthesis of melatonin from tryptophan [35,36,37]. Originally measured by radioimmunoassay and verified by gas chromatography/mass spectrometry (GC/MS), this group successfully identified melatonin in almost all plant tissues, *i.e.*, three tomato cultivars, banana, beetroot, cucumber and the leaves of two tobacco cultivars. The only plant organ in which melatonin was not identified was the potato. Since Dubbels was an avid gardener, all plants studied (except banana) had been organically grown in his garden to reduce the possibility of contamination by melatonin from an extraneous source. The concentrations of melatonin measured varied widely from 5 (in beetroot) to 253 pg/mL (Cultivar Sweet 100 tomato). The authors also mention there were significant variations in the levels of melatonin among samples of the same fruit or vegetable.

Since melatonin had recently been identified as a free radical scavenger [38], Dubbels and colleagues [32] speculated that one function of melatonin in plants was to protect them from ozone (which causes free radical formation) and possibly other oxygen radical generating processes. This was also supported by the observation that the tobacco cultivar most resistant to oxidative damage by ozone also had the highest melatonin levels.

The second seminal report that identified melatonin in higher plants is that of Hattori *et al.* [39]. Their rationale for initiating the studies differed from that of Dubbels and co-workers [32]. Hattori and colleagues [39] knew that in photoperiodically-sensitive mammals, changes in the duration of the nocturnal melatonin peak regulate annual fluctuations in reproductive capability [40,41]. Thus, they reasoned that melatonin in plants may also be involved in influencing seasonal variations in flower growth, *etc.* As further justification for initiating their study, the authors noted that photosynthetic algae also produced melatonin [30].

Hattori and colleagues [39] selected 24 edible plants from 12 families and both dicotyledons and monocotyledons for study, two plant products which duplicated those tested by Dubbels *et al*. [32], *i.e.*, cucumber and tomato. Whereas the concentrations measured in these products varied between the two reports, the reasons for this could have been multiple, e.g., assay variations, time of harvesting the plants, maturation state of the plants, different varieties of the same plant, *etc.* [42]. The method of melatonin estimation employed by Hattori *et al*. [39] was radioimmunoassay, which is generally considered to be less sensitive and specific than the GC/MS technique used by Dubbels and co-workers [32]. As in the first study, the concentrations of melatonin in plant products measured varied widely from 5 pg/g to 5 µg/g tissue.

To further verify their findings, Hattori *et al.* [39] generated concentration displacement curves using authentic melatonin and extracts of several plants. The melatonin curves were not found to be significantly different from those of the extracts of ginger, tall fescue grass, Japanese radish, Japanese ashitaba, rice or shungiku supporting the supposition that the plant extracts contained melatonin. Finally, competition curves using several plant extracts showed that they inhibited iodomelatonin (a ligand for melatonin at membrane receptors) binding to melatonin receptors. Collectively, the findings were consistent with the presence of melatonin in all plant extracts examined.

While the investigations of Dubbels *et al.* [32] (in Germany) and Hattori and colleagues [39] (in Japan) were carried out within the same time frame, they were unaware of each other’s work. There is one common author on the two resulting publications (the primary author of the current review). I did not divulge to either group that a similar study was on-going. This was intentionally done since this field of research was new and I did not want the work of either group to be biased or influenced by the others’ findings, particularly regarding the actual identification of melatonin in higher plants. Each was informed of the others work when the respective papers were accepted for publication.

Following the publication of these reports [32,39], major questions obviously remained unanswered. These included an explanation for the wide range of melatonin concentrations in plants and the functional significance of the indoleamine in plant cells. Moreover, it was unknown whether plants actually synthesized the melatonin they contained or whether it was merely taken up from the growth medium.

## 2. Melatonin in Plant Tissues: Expansion of the Field

In mammals, the tissues that produce gametes and the gamete itself, e.g., the ovum, synthesizes its own melatonin theoretically to protect it from mutilation by toxic free radicals [43]. The assumption is that since the ovum represents the next generation, during maturation, which often is associated with elevated free radical generation, special precautions must be taken to prevent these cells from being oxidatively injured since such damage could lead to the death or abnormal development of the fetus and of the newborn, thereby compromising perpetuation of the species. Similarly, for plants the seeds represent the subsequent generation and if the molecules they contain exhibit excessive oxidative stress the seed may not generate and the next generation would be lost. Also, most seeds are rich in polyunsaturated fats which are rather easily oxidized so potent antioxidants such as melatonin would be important in reducing the likelihood of them being damaged.

While this information was not used as a justification for their study, Manchester *et al.* [44] estimated melatonin concentrations in the seeds (the next generation) of 15 different plants and all had what the authors described as high levels of the indole. These values varied from 2 to 200 µg/g dry weight with the highest values being recorded in the seeds of white and black mustard. In the walnut (*Juglans regia* L.), melatonin levels are in the same range (3.5 µg/g dry weight) [45]. Also of note, the highest levels of melatonin measured in any plant organ to date are in the pistachio nut (*Pistacia vera*) where the reported values are in the range of µg/mL methanolic extract [46]. Obviously, seeds and plants in general contain melatonin levels that greatly exceed those measured in vertebrate blood [47,48] or tissues [49,50,51], where concentrations are usually in the pg/mL and pg/g protein range, respectively. At least by the standards in mammalian tissues, melatonin levels in plant organs are indeed much higher.

As noted, the pistachio kernel has uncommonly high melatonin levels. This finding is of special interest given that the pistachio is a desert plant and survives long periods without water (drought). Moreover, this plant is highly tolerant of salty soils and can survive environmental temperatures ranging from −10 °C to 48 °C. As will be discussed later in this review, each of these circumstances, *i.e.*, drought, saline exposure and extreme temperatures, upregulates melatonin production in plants. Hence, it is possible that the pistachio tree is able to survive and thrive under desert conditions because of its high melatonin levels. On the basis of this, desert plants in general may have high levels of melatonin, e.g., cacti and the desert sage, marigold, lily and willow tree (*Chilopsis*), *etc.* Likewise, melatonin levels may also relate to the regional distribution such that plants that normally grow at the extremes of latitude may have elevated concentrations of melatonin, which may also aid in their survival under harsh, cold conditions (e.g., plants growing on the tundra). We also predict, for the same reason, that extremophiles (organisms which thrive under physically or geochemically extreme conditions) are possibly melatonin enriched relative to the levels of the indole in mesophils or neutrophils.

So-called Chinese/Japanese herbs have historically been touted as having medicinal properties and several are approved for these uses in Canada. Assuming that melatonin, if present in these products, could contribute to their usefulness as treatments for various ailments, prompted Murch *et al.* [52] to estimate levels of the indoleamine in green and golden leaf varieties of feverfew (*Tanacetum parthenium*), St. John’s Wort (*Hypericum perforatum*), Huang-qin (*Scutellaria baicalensis*), and Tanacet, a commercial preparation of feverfew that comes in a tablet form. Tanacet meets the Canadian requirements for use as a medicine. The methodology for extraction and measurement of melatonin used by Murch and colleagues [52] was that described by Poeggeler and colleagues [53].

The feverfew specimens were either fresh, freeze-dried or oven-dried and all were reported to have melatonin levels in the 1–3 µg/g tissue range. Tanacet, the tablet preparation of feverfew, contained significantly less melatonin (0.5 µg/g) than did the levels of this plant. The St. John’s Wort flowers had much higher melatonin concentrations (4.4 µg/g) while the leaves had levels of 1.8 µg/g. *Scutellaria baicalensis* leaves had exceptionally high melatonin values at about 7 µg/g fresh leaves [52].

If the measures are valid, these medicinal herbs contain much higher melatonin levels than do the edible plants studied by Dubbels *et al.* [32] and Hattori and colleagues [39]. The authors concluded that melatonin in these alleged medicinal products may contribute to their anecdotal beneficial physiological effects [52].

**Figure 1:**
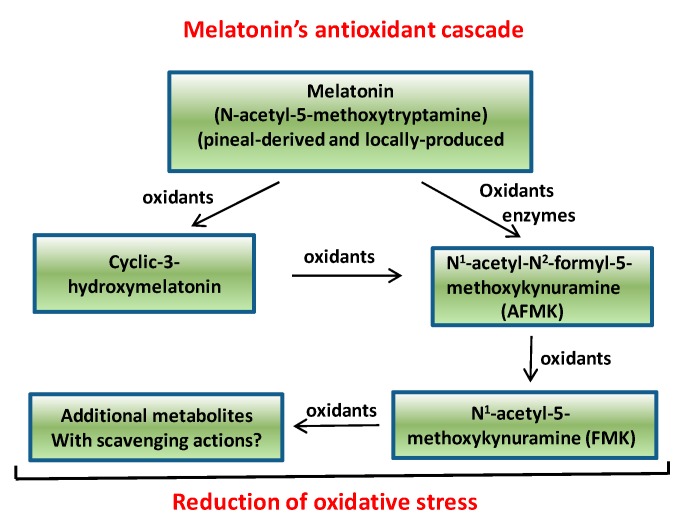
This figure illustrates what has come to be known as the melatonin antioxidant cascade. When it functions in the scavenging of free radicals, melatonin generates the metabolites indicated, all of which are also radical scavengers. This series of reactions greatly increases the ability of melatonin to limit oxidative stress.

As a follow-up to the study of Murch *et al.* [52], using a solid phase extraction method coupled with high performance liquid chromatography/mass spectrometry, Chen *et al.* [54] estimated melatonin levels in 108 Chinese medicinal herbs. Again, their rationale for the study was that, if melatonin is present in significant amounts, it may be a contributing factor to the observed beneficial effects of these preparations. The products from which the extracts were prepared included portions of flowers, seeds, leaves, roots and stems and all met standards for use in traditional Chinese medicine. The majority of herbs tested contained detectable amounts of melatonin of which 64 had values that exceeded 10 ng/g dry mass and several had levels greater than 1 µg/g. The results of this seemingly carefully done study lends credence to the possibility that melatonin in these products could contribute to their medicinal value. This group was especially interested in the possibility that melatonin-containing herbs could be used as a potential treatment of diseases that have a major free radical component. Melatonin, as well as a variety of its by-products that are formed when melatonin detoxifies free radicals, all function in the reduction of oxidative stress [38,55,56,57,58,59,60,61,62] (Figure 1). In neither the Murch *et al.* [52] nor the Chen and co-workers [54] studies, however, did the authors provide evidence that when these herbs are consumed, they change blood levels of the indoleamine.

The initial reports related to the presence of melatonin in higher plants did not attract the investigative efforts of a large number of plant biologists for the first decade or more after the initial reports appeared. During this ten-year interval, however, a number of mostly brief reviews appeared that summarized the early descriptions of the indole in plants, pointed out some of the possible deficiencies in the reported measurements and, importantly, speculated on the functional relevance of melatonin in plants [63,64,65,66,67,68,69,70,71,72]. In general, more credence was given to those reports in which gas chromatography/mass spectroscopy was used to estimate melatonin levels or to verify the reliability of other assays. Also, considering that melatonin may be unstable in plant extracts may have caused some of the measured values to be underestimates of the actual melatonin concentrations in different plant species.

## 3. Melatonin Rhythms in Plants

In the blood of mammals, melatonin levels exhibit a rhythm with low values during the day and high levels at night [73,74,75,76,77]. Since this is common to all mammals regardless of their locomotor activity pattern, melatonin has been referred to as the hormone of darkness or the chemical expression of darkness [78]. This diurnal pattern of fluctuating melatonin levels in animalis perturbed if light exposure occurs at night [79,80,81] while in constant dark conditions the rhythm persists, *i.e.*, the rhythm is truly circadian.

Considering the ubiquitous nature of the blood melatonin rhythm in mammals, there was also interest in whether similar variations existed in plants and if melatonin was involved in photoperiodism in these species. Using a weed (*Chenopodium rubrum*; syn, *Oxybasis rubra*; red goosefoot and other common names) that commonly grows in temperate climates in many areas of the world, Kolar *et al.* [82] found that in this species nighttime levels of melatonin exceeded those measured in plants harvested during the day. Thus, in this plant, as in mammals, the melatonin rhythm could provide information regarding night length. In contrast to these observations, Tan *et al.* [83] using the water hyacinth (*Eichornia crassipes (Mart) Solms*) found that melatonin levels do exhibit a rhythm but peak levels occurred near the end of the light period rather than at night. Thus, in this plant species melatonin clearly does not serve as a messenger of darkness. Also, daytime melatonin levels were higher when the plants were grown under sunlight (10,000–15,000 µW/cm^2^) compared to those grown indoors under artificial light (400–450 µW/cm^2^). In addition to the observed melatonin rhythm, there was a similar cycle of N1-acetyl-N2-formyl-5-methoxykynuramine (AFMK); this product peaked shortly after that of melatonin. Since AFMK is a metabolite of melatonin when it scavenges free radicals (Figure 1), we surmised that melatonin functions as a radical scavenger in the water hyacinth as in animals [84,85,86,87,88,89]; this accounted for the rise in AFMK shortly after that of melatonin. That melatonin functions as a free radical scavenger in this plant is also supported by the observation that melatonin concentrations were much higher in plants grown under sunlight than under much dimmer indoor light. The outdoor light intensity causes highly elevated photosynthetic activity, a process that generates free radicals [90]. Consequently, the rise in free radicals presumably initiated a compensatory increase in melatonin to protect the plant from oxidative stress.

A third rhythmic pattern of melatonin was described by Tal *et al.* [91] in the green macroalga. The Genus of the alga was *Ulva*, but the species could not be identified, which is typically difficult to do for green algae [92,93]. Even when this species was grown as a free floating culture under constant photoperiod and water levels, it exhibited a semi-lunar rhythm in melatonin concentrations that correlated with the spring tides. These workers speculated that the elevated levels of melatonin at the time of predicted low tides were for the purpose of protecting the alga from increased oxidative stress that would normally occur due to the low water levels. During this interval, algae are subjected to elevated temperature, desiccation and changes in salinity all of which would cause stress. Thus, like Tan *et al.* [83] in the water hyacinth, Tal and co-workers [91] measured a presumptive endogenous rise in melatonin that occurs as a consequence of a natural environmental stress. Since this rhythm persisted under constant conditions, however, during evolution the cycle was seemingly incorporated into the genetic machinery of the plant. Alternatively, the melatonin rhythm in this alga may not be inherent but rather induced by the gravitational pull of the moon.

Tart cherry (*Prunus cerasus*) is the first fruit in which melatonin levels were comprehensively studied [94]. The two varieties tested, *i.e.*, Montmorency and Balaton, contained markedly different melatonin concentrations [13.4 µg/g FW (fresh weight) and 2.1 µg/g FW, respectively]. Considering that melatonin is a sleep promoting agent [95,96], the authors of this report suggested that consumption of cherries or especially the intake of cherry juice concentrate (which contains much higher melatonin levels than the cherries themselves) may improve sleep quality. Subsequent publications related to this suggest that cherry products may be useful for improving sleep quality, particularly in the elderly [97,98,99,100,101].

Finally, in at least two varieties of cherry fruits melatonin levels vary over a 24 h period and also with fruit development [101]. In “Hongdeng” (*Prunus avium* L. cv. Hongdeng) and in “Rainier” (*Prunus avium* L. cv. Rainier) cherries the 24 h pattern of melatonin exhibited two peaks. The nighttime peak occurred at roughly 05:00 h and in the day the peak was in the late afternoon, corresponding to the highest temperature and light intensity. The highest melatonin levels in the “Hongdeng” cherry were about 7.7 µg/g FW while in “Rainier” the values approached 20 µg/g FW. The 24 h measurements were performed on fruit during Stage 1 of development (shortly after the flower falls with quick fruit expansion and rapid growth of the pit and endosperm).

**Figure 2:**
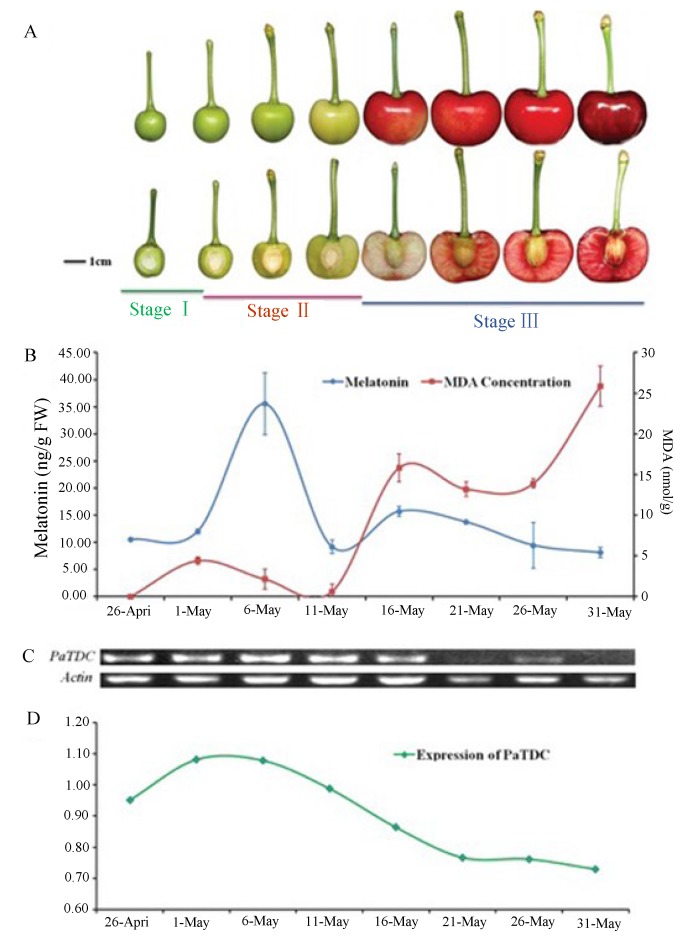
The association of melatonin, malondialdehyde (MDA) and the expression of PaTAC, the gene that encodes tryptophan decarboxylase, the potential rate limiting enzyme in serotonin synthesis in plants. (**A**) Intact and hemisectioned “Hongdeng” cherry fruits in different stages of development; (**B**) The levels of melatonin and the lipid peroxidation product, MDA, during fruit development; (**C**) PaTAC expression in cherry fruits during development; (**D**) Expression of PaTDC. From Zhao *et al.* [101] with permission.

For both “Hongdeng” and “Rainier” cherries, melatonin levels also varied markedly with the developmental stage of the fruit (Figure 2). The melatonin levels were highest at Stage 2 of development (period of embryo development and endocarp lignification). Peak melatonin levels at this time were again higher in “Rainier” than in “Hongdeng” cherries (roughly 125 *versus* 36 µg/g FW). During Stage 3 of fruit development (fruit expansion and peel and fruit coloration) melatonin levels remained low and were inversely correlated with the levels of peroxidized lipids in the fruit. Because of the inverse relationship of melatonin to lipid peroxidation (in Stage 3) and the very high levels during Stage 2 (when free radical generation is maximal), Zhao *et al.* [101] concluded that melatonin functions as an antioxidant in the cherry fruit.

A similar relationship between apple (*Malus domestica Borkh. cv. Red*) maturation and melatonin levels was reported by Lei and colleagues [102]. As in the cherry, highest melatonin levels co-existed with the periods of the most rapid expansion and increased respiration in the apple, points at which free radical generation is maximal.

Again, there was also an inverse association between melatonin concentrations and the amount of peroxidized lipids. In addition to documenting that the concentration of melatonin changes dramatically during apple development, this is the first study to identify melatonin in this fruit. Clearly, there are changes in the melatonin concentrations as plants mature as in animals. Whether any of these fluctuations are truly endogenous melatonin rhythms seems questionable (with the possible exception of the tide-correlated cycle in macroalga [91]). As summarized below, there are a variety of factors that impact the melatonin synthetic activity of plants and the rhythms observed, for example, the cyclic changes noted by Kolar *et al.* [82] and Tan and colleagues [83], were likely related to environmental changes over the 24 h period which enhanced the number of free radicals formed while those reported by Zhao and co-workers [101] and Lei *et al.* [102] were probably driven by intrinsic metabolic processes that also induced free radical generation.

## 4. Effect of Stress on Plant Melatonin Synthesis

Both abiotic and biotic stresses have been shown to induce melatonin synthesis in plants. The compensatory rise in melatonin under stressful conditions, in turn, helps to alleviate the negative consequences of stress, in particular oxidative stress, in the plants. Typically, stress of any type to plants compromises their growth which is often secondary to excessive generation of reactive oxygen species (ROS) including free radicals. A well-known feature of melatonin in animals, and also in plants, is the high efficiency by which it nullifies the adverse effects of toxic oxygen derivatives due to its ability to neutralize them [38,58,103,104]. The presence of melatonin increases the stress resistance of plants which aids in their ability to survive and thrive.

**Figure 3:**
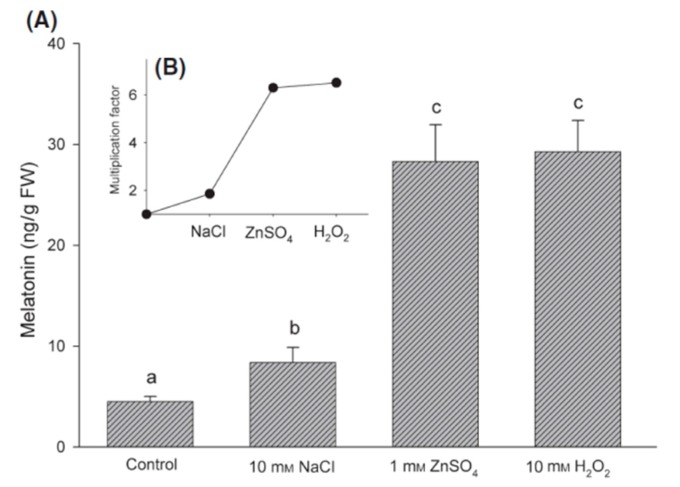
(**A**) Increase in melatonin levels in barley roots after exposure to various treatments for 72 h; (**B**) Fold increases relative to control value. FW = fresh weight. Different letters indicate statistically significant differences (*p* < 0.05). From Arnao and Hernandez-Ruiz [105] with permission.

Some of the abiotic stresses shown to augment oxidative stress in plant cells include soil and air pollutants, cold and hot temperatures, high light intensity, *etc.* Arnao’s group has examined a variety of stresses relative to their ability to impact melatonin levels in barley roots [105] and in lupin [106]. In the former study, chemical stresses including NaCl, ZnSO_4_ or H_2_O_2_ were applied to barley roots and, after 72 h, melatonin was assayed in the whole plant. Each of these challenges induced significant rises in melatonin levels with ZnSO_4_ and H_2_O_2_ having greater stimulatory effects than NaCl (Figure 3) [105]. These responses were both toxin dose and time-dependent. The upregulated melatonin levels, in turn, protected the plant from these chemical agents.

Soil salinization is often a result of irrigation, a process that is common in many parts of the world [106,107]. The high salt content causes plants to lose vigor; however, by means of their ability to upregulate melatonin synthesis they are more tolerant of salty contaminants. It is possible that genetically engineering plants to produce elevated melatonin levels, a feat that has already been accomplished [108,109], may permit them to be grown in increasingly salt-contaminated soils.

Li and colleagues [110] specifically examined the ability of supplemental melatonin to protect a plant from induced saline stress. In this study, 10-day-old apple (*Malus hupehensis*) seedlings under hydroponic conditions were stressed with salt (100 mM) with or without the addition of melatonin (1 µm). The high saline-stressed plants suffered with stunted growth, reduced rate of photosynthesis and depressed chlorophyll levels all of which were alleviated when melatonin was also added to the growth medium. Melatonin also limited oxidative stress by reducing H_2_O_2_ levels and stimulated antioxidative enzymes (catalase, peroxidase, and ascorbate peroxidase). The promotion of antioxidative enzyme activities is a well-known function of melatonin in animal cells as well [111,112,113]. These stimulatory actions of melatonin are generally believed to involve cell membrane receptors [114] for the indole, structures that have yet to be identified in plants. Finally, Li *et al.* [110] also measured the expression of ion channel genes, *MdNHX1* and *MdAKT1*, and found them to be upregulated in the leaves of the apple seedlings treated with melatonin; this presumably aided the plants in maintaining ion homeostasis.

Melatonin also has been used in combination with ascorbic acid (AsA) to protect bitter orange (*Citrus aurantium* L.) seedlings from a high salt environment [115]. *Citrus aurantium* received significant public attention over a decade ago when extracts of this fruit were espoused as weight-reducing agents. The negative cardiovascular side effects of the extracts, however, have rendered them of little interest currently in terms of weight loss although they have industrial uses not related to their consumption.

In the report of Kostopoulou and co-workers [115], bitter orange seedlings were grown under greenhouse conditions in soil contaminated with 100 mM NaCl for 30 days. Some of the plants were irrigated every 3 days with water containing 0.50 mM vitamin C (AsA) and 1 µM melatonin. This combination had highly significant protective actions against saline toxicity including reduced NaCl-mediated electrolyte leakage, depressed lipid peroxidation and lowered pigment degradation. The AsA/melatonin treatment benefits in the NaCl_stressed plants was also apparent in the regulation of *CaMIPS*, *CaSLAH1* and *CaMYB73* expression; these genes relate to sugar metabolism, ion homeostasis and transcription regulation. Collectively, the changes induced by the combination of AsA and melatonin provide evidence of increased stress tolerance in *Citrus aurantium* [116]. What are particularly noteworthy about this study are the remarkably different concentrations of AsA and melatonin that were used to protect against saline toxicity, *i.e.*, 0.50 mM and 1 µM, respectively.

The only study to examine the immunocytochemical localization of melatonin and serotonin in a plant was performed by Mukherjee *et al*. [117]. The study revealed that these compounds are differentially distributed in the primary roots of sunflower (*Helanthus annuus*) seedlings. When exposed to 120 mM NaCl both serotonin and melatonin levels increased at these sites. Salt exposure also caused a 72% rise in HIOMT activity in sunflower seedling cotyledons. Since this enzyme is responsible for melatonin formation, its altered activity likely explains the increased melatonin levels. However, since melatonin synthesis in the animal pineal gland typically depletes serotonin levels in contrast to the present study where they were elevated under salt stress, the increase in this constituent obviously required an independent process mediated by NaCl.

Since melatonin is a highly efficient direct free radical scavenger and indirect antioxidant (due to its ability to stimulate antioxidative enzymes), the authors of the saline contamination studies put significant emphasis on the idea that melatonin’s beneficial actions are probably attributable to its ability to resist oxidative stress. Elevated salinity, however, has other negative actions in plants that seem unrelated to free radicals [118,119]. Thus, the precise mechanisms that melatonin utilizes to preserve plant survival against the toxicity of high levels of saline is yet to be fully defined.

Heavy metals are becoming progressively more common as soil pollutants in industrialized areas. Often the contaminated soils are placed “off limits” or they are moved to another location where they are less damaging. As an alternative, growing green plants in the contaminated soil which take up and tolerate the pollutants is an accepted means of cleaning soil, a process referred to as phytoremediation [120]. Tan *et al.* [121] tested whether the addition of melatonin to soil contaminated with a heavy metal (copper) would also be taken up by a pea plant thereby rendering it more resistant to the absorbed toxic metal; if so, it could make the plant a candidate for phytoremediation. In a pilot study, this group showed that growing pea plants (*Pisum sativum*) grown in a soil contaminated with copper exhibited increased tolerance to the metal when the soil also contained melatonin. This proof-of-principle investigation indicated that melatonin enriched plants could have a significant phytoremediation capacity.

The publication of Tan and colleagues [121] not only showed that supplemental melatonin may be useful in enhancing the phytoremediative capacity of plants but, additionally, demonstrated that the indole obviously protects the pea plant when exposed to elevated concentrations of copper in the soil. This is supported by other publications as well (see below).

The exposure of *Ulva* (green macroalga) to heavy metals for eight days caused a marked rise in melatonin levels in this plant [91]. The metals used included lead (5 mg/L), zinc (30 mg/L) and cadmium (1 mg/L) and were merely added to the water in which the alga was grown. The most marked increase in melatonin (171% rise) occurred in alga exposed to cadmium even though its concentration was less than that of either lead or zinc. All three metals also induced an elevation in tissue antioxidative enzyme activity (superoxide dismutase; SOD), a change indicative of an oxidative challenge.

Even though cadmium exposure stimulated a highly significant rise in endogenous melatonin levels in *Ulva*, chlorophyll concentrations exhibited a inverse correlation and dropped as a result of metal exposure. When additional exogenous melatonin (50 mg/L) was added to the water, however, it relieved the oxidative stress such that the reduction in chlorophyll levels was attenuated. High water temperatures (30 °C or 37 °C *versus* 23 °C) also increased endogenous melatonin concentrations in *Ulva* in a temperature-dependent manner. Both heavy metal exposure [122,123] and elevated temperature [124] are known to induce oxidative stress in plants.

On the basis of their results, Tal *et al.* [91] proposed that melatonin provides a major, and perhaps even the primary defense mechanism in *Ulva* against oxidative stress. Although no aspect of melatonin synthesis was evaluated (e.g., enzyme activities) in this study, the rises in melatonin were very likely a consequence of its *de novo* synthesis. The authors also suggest that this protective mechanism against oxidative stress may be shared by many intertidal organisms.

## 5. Melatonin Protects Plants from Abiotic Stresses

One consequence of elevated greenhouse gases is a change in weather patterns that occur over extended periods of time, which then impact plant ecosystems. These climate changes include warmer or colder temperatures and changes in water availability due to frequent storms or intermittent drought [125,126,127]. If they are to survive, plants must adapt to these changes. Melatonin is one molecule that seems to aid them in resisting some of the consequences of marked changes in their environment.

Drought causes the loss of a significant portion of many commercially-important crops. That melatonin has a role in interfering with the ability of water restriction to compromise plant physiology was first documented by Wang *et al.* [128]. They reduced, by 50%, the amount of water supplied to one-year-old greenhouse-grown “Hanfu” apple trees (*Malus domesticus* Bokh.) with half of the plants receiving a 100 µM melatonin solution which was added to the growth medium when water was given. The consequences of drought were monitored by evaluating leaf senescence.

Drought accelerated the appearances of the senescent changes in the leaves of the apple trees. These perturbations included increased chlorophyll degradation, upregulation of *senescence-associated genes* and of *pheophorbide a oxygenase*; these contributed to the inhibition of photosynthesis and a reduction in the efficiency of Photosystem II. Uniformly, these changes were reduced or totally prevented in the melatonin-supplemented trees. Since hydrogen peroxide was quenched and the activities of antioxidant enzymes as well as the capacity of the ascorbate-glutathione cycle were enhanced as a result of melatonin treatment, Wang *et al.* [128] confidently concluded that melatonin’s function as an antioxidant accounted for its protective actions against drought.

In a subsequent report, a different means was used to prove that melatonin increased drought tolerance in plants [129]. In this study, MicroTom tomato plants (three lines) overexpressing ovine HIOMT and ovine AANAT were compared to wild type plants in terms of their responses to drought. When subjected to water restriction for 20 days, both the wild type and transgenic plants exhibited significant wilting; however, 48 h after being re-watered the transgenic plants had almost totally recovered while the control plants, which lacked the ability to synthesize extra melatonin, remained severely wilted.

In studies using *Arabidopsis* exposed to cold ambient temperature, which stimulates endogenous melatonin synthesis (like water restriction), genes related to drought, *i.e.*, *Drought Response Element Binding* factors (*DREBs*), are upregulated [130,131]. It is likely that these were also upregulated in the two previous studies where melatonin was used to enhance drought tolerance and, as a result, contributed to melatonin’s ability to diminish the damaging effects of water restriction.

Grapes *per se* and the products they produce are obviously major economic items in many regions of the world. Grape plants are typically cultivated in semi-arid regions with the soil being well drained. Because of this, grape plants are often subjected to drought conditions and any metabolic process that would effectively increase the drought resistance of grapes, as with many other plants, would be of critical interest to the viticulture industry. Meng *et al*. [132] performed a study using cuttings from Brown one-year-old Riesling (*Vitis vinifera*) grape plants to determine whether the indole would be beneficial during drought. The cuttings were grown in a combination of garden soil, vermiculite and humus subjected to polyethylene glycol (PEG)-induced water deficient stress for 12 days with or without melatonin supplementation. In the absence of melatonin, the water deficiency severely limited the growth of the cuttings, induced oxidative damage, as measured by the rise in malondialdehyde levels, due to O_2_•- and H_2_O_2_ generation, and compromised the potential efficiency of Photosystem II while depressing chlorophyll concentrations. The application of melatonin attenuated all of the changes observed after PEG application alone. Importantly, melatonin aided in the preservation of chloroplast morphology as evidenced by their ultrastructural appearance. The authors [132] attributed the protective effects of melatonin to its direct free radical scavenging actions and to its indirect effects in stimulating antioxidant enzymes, which are also well-known actions of melatonin in animals [113]. The preservation of chloroplast morphology, efficiency of Photosystem II and preserved chlorophyll were also critical findings that will likely have applications in the viticulture industry.

**Figure 4:**
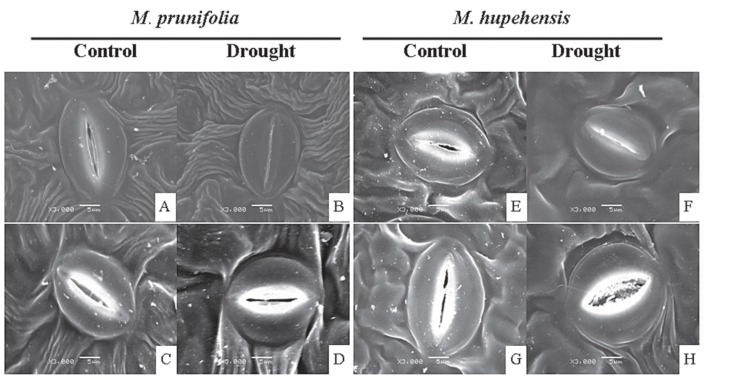
Scanning electron micrographs of stomata of leaves from *M. prunifolia* and *M. Hupehensis* plants. **A** and **E** = open stomata of control leaves; **B** and **F** = closed stomata from leaves subjected to 5 days of drought; **C** and **G** = open stomata of control leaves after treatment with 100 µM melatonin; **D** and **H** = open stomata of leaves from drought-stressed plants pretreated with melatonin. From Li *et al.* [133] with permission.

As with grapes, an insufficient water supply compromises apple production secondary to perturbations in plant physiology. In view of earlier studies related to melatonin and drought, Li and co-workers [133] tested whether melatonin would also mitigate low water-mediated changes in drought-resistance (*Malus prunifolia*) and drought-sensitive (*M. hupehensis*) apple plants. In both apple tree strains, melatonin conserved water in the leaves, reduced electrolyte leakage, limited chlorophyll loss and improved photosynthesis under drought conditions. In plants, drought is known to upregulate the production of abscisic acid (ABA) which leads to the closure of the stomata and the expression of drought stress-related genes [134]. Melatonin downregulated ABA synthetic gene (*MdNCED3*) and upregulated its catabolic genes (MdCYP707A1 and MdCYP707A2) and, as a result, reduced ABA levels in drought-stimulated apple plants. Melatonin also functioned as a scavenger of H_2_O_2_ and stimulated enzymes which metabolized H_2_O_2_ to non-harmful products. This combination of effects caused the stomata to remain open. Regulation of stomatal opening/closure is an important means by which plants regulate water balance and resist drought (Figure 4) [135,136]. Again, the authors surmised that these findings could make melatonin application an attractive means to reduce the consequences of drought in apple plants.

The data from the publications related to the ability of melatonin to improving drought tolerance are compelling and provide unequivocal evidence related to its benefits to plants under restricted water conditions. Whereas these studies did not specifically examine the ability of melatonin to improve crop yield, based on the findings discussed below, this would be a reasonable assumption.

As a follow-up to the observations of Hattori *et al.* [39] who showed that grass (tall fescue) contains melatonin, Shi *et al.* [137] examined the functional significance of melatonin in bermudagrass (*Cynodon dactylon* (L). Pers.). Bermudagrass is of high economic importance since it is cultivated throughout the world as turf grass for lawns, recreation parks and sports fields. In addition to protecting the grass from abiotic stresses, melatonin clearly had beneficial metabolic actions in this plant as well. To test the ability of melatonin to overcome the effects of drought stress, 21-day-old bermudagrass plants were irrigated with water or with solutions containing melatonin (4 µM, 20 µM or 100 µM) for 7 days. Thereafter, for an additional 21 days some plants were subjected to drought conditions, cold temperatures (4 °C) or to high salinity (up to 300 mM).

Under each of these stresses, supplementation with melatonin improved survival and the percentage of plants that survived generally correlate with melatonin’s concentration levels in the grass leaves. Irrigating the plants with melatonin also enhanced the chlorophyll levels and improved the height and biomass of the grass plants. Plants pre-treated with melatonin also exhibited enhancement of 54 different metabolites that were perturbed by the stresses imposed. Genome-wide transcriptomic evaluation identified nearly 4000 transcripts that were differentially expressed as a consequence of melatonin exposure. Based on the large number of beneficial effects of melatonin that was measured in bermudagrass, the authors also surmised that melatonin had a major impact on the nitrogen and carbohydrate metabolism and on transcriptional reprogramming [137].

Weeda and colleagues [138] also performed a genome wide analysis of *Arabidopsis* plants following their growth in melatonin solutions. The plants were grown for 16 h in solutions containing either 100 pM or 1 mM melatonin. The lower levels of melatonin had what the authors considered a minor response with 51 genes being downregulated and 30 being upregulated. However, the 1 mM dose caused upregulation of 566 while 742 genes were downregulated. The bulk of the stimulated genes related to plant stress defenses while genes linked to auxin responses and signaling were associated with the synthesis of cell wall components were downregulated [138].

As shown in studies summarized above [129,132,134] and in the report by Shi and colleagues [137], melatonin not only protects against deterioration during drought stress, but also helps plants to recover after drought has occurred and water is resupplied. Obviously, the ability to adapt to intermittent periods of severe water restriction would be an aid in helping any plant to survive.

Ambient temperature can fluctuate dramatically throughout the growing season of plants. Thus, they are often exposed to rapidly elevated or depressed temperatures which they must tolerate if they are to thrive. Using melatonin concentrations in the mM range, Lei *et al*. [139] reported that the indole significantly suppressed cold-mediated apoptotic cell death in a carrot (*Daucus carota* L.) suspension. In this study, the ability of melatonin to inhibit apoptosis seems unrelated to its radical scavenging actions; rather, since melatonin significantly stimulated putrescine and spermidine levels in carrot cells, it was hypothesized that the actions of melatonin on polyamines improved carrot cell survival.

In an *in vivo* study, Posmyk and colleagues [140] used cucumber seeds (*Cucumis sativus* L. var. Odys) to confirm the protective actions of melatonin against reduced environmental temperature. Incubating seeds in a melatonin solution increased their germination at 10 °C and reduced the degree of lipid peroxidation in cucumber cellular membranes. As in a previous study that used melatonin as an antidote to copper toxicity [121], excessively high melatonin levels actually aggravated cold temperature-mediated oxidative damage to cucumber cellular proteins.

**Figure 5:**
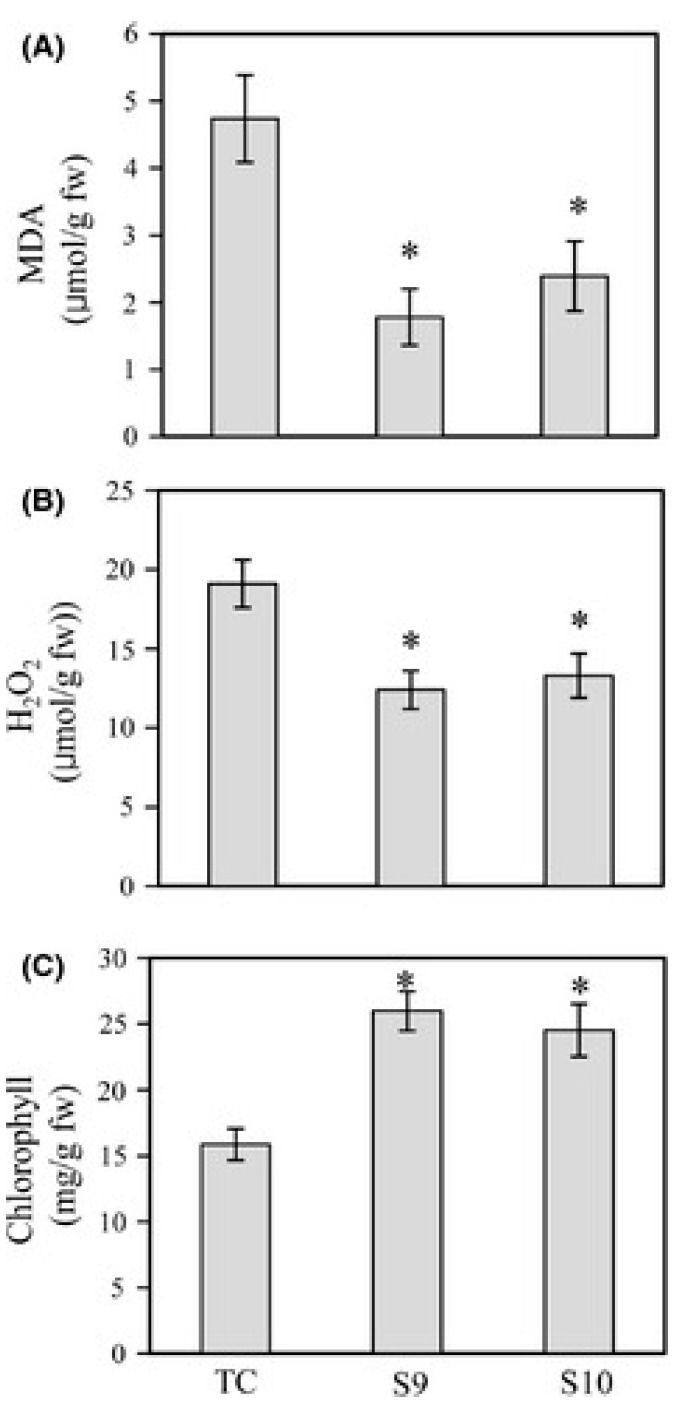
Levels of (**A**) lipid peroxidation products (malondialdehyde); (**B**) hydrogen peroxide (H_2_O_2_); and (**C**) chlorophyll in butafenacil-treated rice seedlings containing normal melatonin levels (TC) and in transgenic rice seedlings containing elevated melatonin concentrations (Supplementary S9 and S10). Higher melatonin levels reduced lipid peroxidation and H_2_O_2_ production and elevated chlorophyll levels. * *p* < 0.05 *versus* TC. From Park *et al.* [141] with permission.

Melatonin-enriched transgenic rice plants (*Oryza sativa* cv Dongjin) exhibited increased resistance to the herbicide, butafenacil, an agent known to generate the reactive singlet oxygen. Melatonin-rich seeds and controls were germinated and grew equally well under constant dark conditions [141]. When these etiolated plants were transferred to a light environment, however, the control plants underwent necrosis while those containing genetically-enhanced melatonin levels survived and exhibited butafenacil-resistant phenotypes. These seedling which contained higher melatonin concentrations also had elevated chlorophyll levels (Figure 5) and lower malondialdehyde concentrations. This is the first study to show that melatonin protects any plant from the toxicity of an herbicide, in this case one that stimulates the production of an active oxygen metabolite known to inflict oxidative damage. This finding is similar to those in animals [142,143], where herbicides are also toxic via similar mechanisms, *i.e.*, the generation of ROS and prevention by the application of melatonin.

High intensity ultraviolet (UV) light generates free radicals in animal and plant tissues when they are exposed to this radiation [144]. When *Glycyrrhiza uralensis* plants were exposed to high intensity UVB light, melatonin levels increased in the roots several-fold; the authors speculated that the rise in melatonin was for the purpose of protecting plant tissues from oxidative stress caused by the free radicals induced by UVB exposure. This particular plant was used as an experimental model because of its antiviral and antitumor medicinal properties.

UV radiation is becoming an increasingly acute problem with the breakdown of the ozone layer in the lower stratosphere which allows these wavelengths, which were previously filtered, to strike the Earth in greater abundance. Based on the findings reported by Afreen and co-workers [144], it seems likely that plants that inhabit terrain under the ozone hole may possess higher endogenous levels of melatonin than the same plant grown elsewhere. Using the same rationale, it is also possible that plants growing at very high altitudes where UV exposure is also elevated, may have increased melatonin concentrations as a means of protection.

As reviewed above, a report by Tan and co-workers [121] showed that melatonin may be an aid for improving the phytoremediative capacity of plants. A more complete study in which melatonin was shown to defend against copper toxicity was published by Posmyk *et al.* [145] using red cabbage seedlings (*Brassica oleracea rubrum*). In this case, cabbage seeds were improved with one of three melatonin concentrations (1, 10 or 100 µM) and after germination the seedlings received copper (as 0.5 mM CuSO_4_) in water.

In seeds incubated with either 1 or 10 µM melatonin, copper-mediated oxidative stress, as indicated by elevated lipid peroxidation and DNA endoreplication blockade, was significantly reduced. Conversely, the 100 µM melatonin concentration had the opposite effect and enhanced oxidative damage in copper-exposed seedlings. These findings suggest that under some circumstances high levels of melatonin may be toxic to plants.

## 6. Melatonin Protects Plants from Biotic Stresses

**Figure 6:**
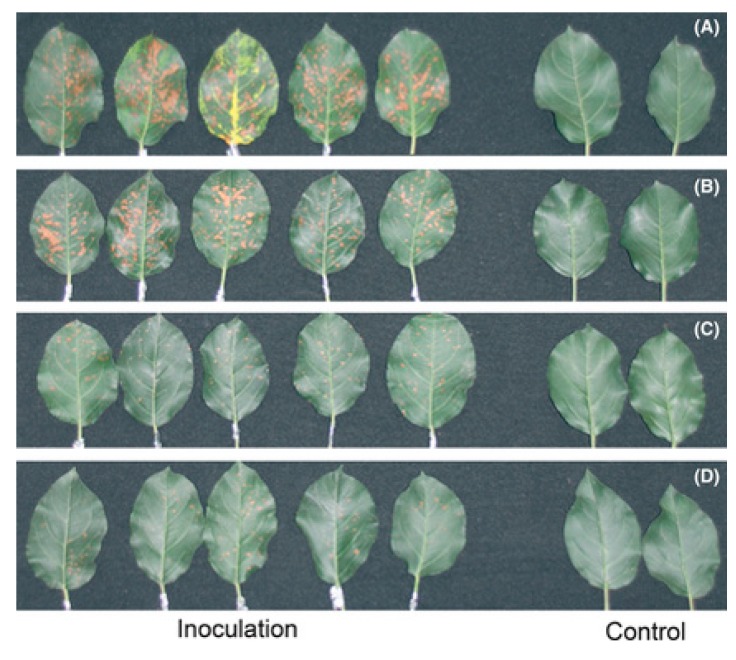
Apple leaf phenotypes at 20 days after being brushed with Marssonina spores; this fungus causes apple blotch disease. Control leaves from trees not brushed with Marssonina spores. Marssonina inoculated leaves from trees that were irrigated with (**A**) no melatonin; (**B**) 0.05 mM; (**C**) 0.1 mM or (**D**) 0.5 mM melatonin solution. From Yin *et al.* [149] with permission.

Marssonina apple blotch is a serious fungal disease propagated by *Diplocarpon mali* [146]. This condition causes apple trees to lose their leaves prematurely which compromises the hardiness of the trees and diminishes fruit yield [147]. The fungus initially attacks the leaves but can spread to the fruit as well [148]. Yin and colleagues [149] used the leaves of *Malus prunifolia* in which to examine the efficacy of melatonin as an inhibitor of Marssonina phytopathology. The authors showed that pretreatment of apple trees with melatonin improved their resistance to fungus-inoculated trees, which without melatonin leaves turned yellow and the expanding leaf spots coalesced (Figure 6). Other measurements including the total number of lesions, the efficiency of Photosystem II, chlorophyll content, intracellular H_2_O_2_ levels and elevated plant defense-related enzymes all support the protective actions of melatonin against this biotic stress. The authors believe the use of melatonin pretreatment may be an effective strategy to curtail Marssonina fungal infection in *N. mali*. Mechanistically, how melatonin ensured acquisition of resistance still requires clarification. This is the first report that melatonin is useful in constraining biotic stress in any plant species.

**Figure 7:**
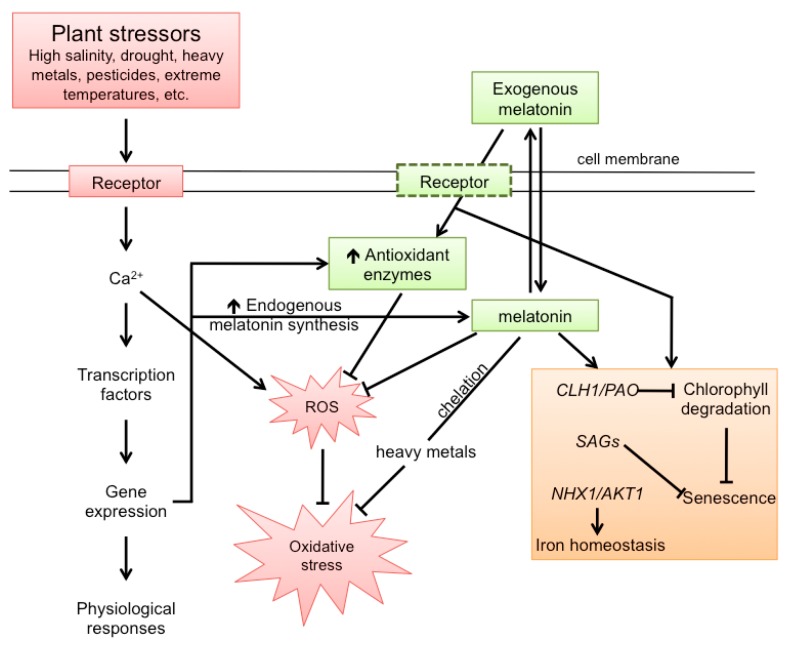
Abiotic stresses negatively impact plant physiology. These stressors induce a cascade of events (left portion of figure) which leads to the elevated production of toxic reactive oxygen species (ROS) and aging. Concurrently, the stresses upregulate antioxidant enzyme synthesis and endogenous melatonin production both of which provide protection against ROS. Additionally, the application of exogenous melatonin also provides protection against ROS and aging. Melatonin receptors (hyphenated box) are included in the cell membrane; however, these receptors have not heretofore been documented. CLH1/PAO = chlorophyll degradation-related genes; NAX1/AKT1 ion channel-related genes; SAGs = senescence associated genes.

A different approach was taken by Lee and associates [150] to examine the efficacy of melatonin in combatting biotic stress. Rather than providing supplemental melatonin, they inactivated the serotonin N-acetyltransferase gene in two *Arabidopsis* T-DNA insertion mutant cell lines which led to a substantial reduction in endogenous melatonin levels in these plants. As a result, the plants also exhibited an elevated susceptibility to the avirulent pathogen, *Pseudomonas syringae* pv. tomato DC3000 harboring the elicitor avrRpt2. The loss of resistance to this pathogen was also associated with reduced inductions of the *PR1*, *ICS1*, and *PDF1.2* genes, which normally provide a defense against the bacterial pathogen. Since melatonin acts upstream of the synthesis of salicylic acid (SA), the depressed melatonin levels in the knockout *Arabidopsis* may have been responsible for the lowered SA levels which, in turn, rendered these plants more susceptible to the pathogen. Figure 7 illustrates the multiple means by which melatonin reduces abiotic stress in plants as illustrated in the previous paragraphs.

## 7. Melatonin and Senescence in Plants

Depending on the species, different external and internal factors determine the rate of foliar senescence, a physiologically regulated process. Leaf death is preceded by chlorophyll degradation, loss of molecular integrity, relocation of nutrients via the phloem, changes in phytohormones (auxin, cytokinins, abscisic acid, ethylene and jasmonic acid), disintegration of petiole cell walls and finally cell death [151,152].

To determine how the senescent processes are influenced by melatonin, Arnao and Hernandez-Ruiz [153] segmented barley leaves (*Hordeum vulgare* L.) and placed them in Petri dishes containing appropriate medium; then, the leaf segments were treated with melatonin or not. Judging from the loss of chlorophyll, leaves treated with melatonin exhibited delayed senescence. The effect of melatonin was concentration dependent (most effective concentration was 1 mM). The mechanisms by which melatonin deferred senescent changes in barley leaves was not determined but may have been a consequence of its free radical scavenging activities or secondary to an inhibition of senescence associated genes [153].

**Figure 8:**
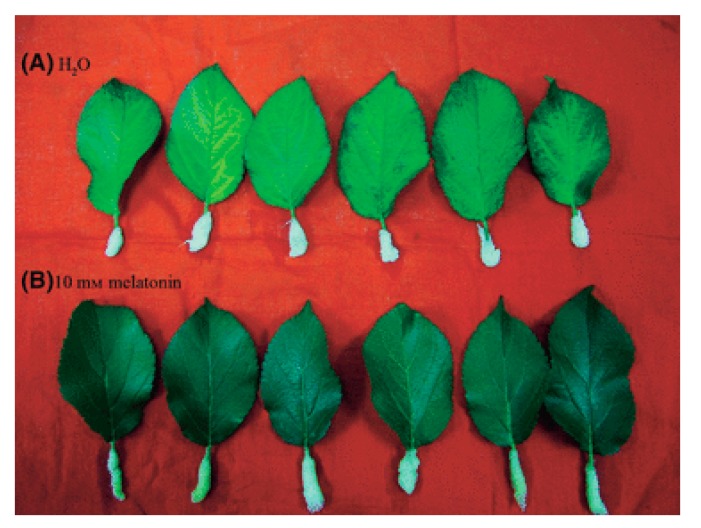
Detached apple leaves treated with (**A**) water only (H_2_O) or with (**B**) water containing melatonin (10 mM melatonin) and kept in the dark for 12 days. Melatonin delayed senescent changes in the leaves as illustrated by the preserved chlorophyll levels. From Wang *et al.* [154] with permission.

In a series of thorough studies, Wang and associates [154,155,156] clarified melatonin’s role in forestalling leaf senescence. When detached apple leaves were kept in the dark to provoke more rapid aging, those treated with 10 mM melatonin lost their chlorophyll slower (Figure 8) and maintained maximal potential Photosystem II efficiency [154]. Moreover, melatonin inhibited gene expression for the key enzyme that degrades chlorophyll (*pheide a oxygenase*) and also inhibited *senescence associated gene 12*, both of which contributed to delayed aging in melatonin-treated leaves. The culprits that caused the observed changes were surmised to be toxic oxygen derivatives since melatonin also suppressed H_2_O_2_ accumulation in the leaves; thus, the ability of melatonin to determine the senescence rate is a consequence of its antioxidant activity.

An *in vivo* study in which apple plants were grown in soil supplemented regularly with melatonin also activated changes consistent with delayed aging [155]. The authors compared a gamut of metabolic endpoints in the leaves of control and melatonin-treated plants. It was shown that melatonin delayed protein degradation, maintained significantly higher Photosystem II activity as measured by preserved chlorophyll levels as well as three photosynthetic end products (sorbitol, sucrose and starch). Melatonin-treated leaves also had improved nitrogen, total soluble protein and Rubisco protein concentrations. This study clearly reveals the large number of positive metabolic actions melatonin has in plants, all of which could contribute to its ability to delay senescence.

Wang *et al.* [156] added an extra level of refinement to their studies related to the role of melatonin in apple leaf aging by performing a proteomic analysis of leaves undergoing natural aging *versus* those aging more slowly due to melatonin treatment. A GO analysis of Blast2GO showed that of the hundreds of proteins altered by melatonin, they were primarily located in the plastids. In general, melatonin downregulated proteins that are typically upregulated during the senescence process. This is certainly the most thorough study related to the action of melatonin on protein metabolism in any plant and the results contribute information on the mechanisms by which melatonin delays aging in plants.

A detailed analysis of some of the genes involved and the measurement of melatonin levels associated with the development of *Arabidopsis* was recently published [157]. Additionally, these workers defined the role that exogenously-applied melatonin has on rosette leaf senescence. During plant development, and particularly in the latter stages (40–60 day old plants), endogenous melatonin levels increase rapidly from 0.5 ng/g FW at day 30 to 2.0 ng/g FW by day 60. When 60-day-old *Arabidopsis* plants were treated with additional exogenous melatonin, leaf senescence was delayed as indicated by the preserved chlorophyll levels. Also, when plants were supplemented with melatonin, the expression level of *AUXIN RESISTANT 3 (AXR3) INDOLE-3-ACETIC ACID INDUCIBLE 17 (IAA17)* was significantly downregulated. The downregulation of IAA17 by melatonin may have caused a drop in the expression of *SENESCENCE 4* and *SENESCENCE-ASSOCIATED GENE 12*. The results show that the gene, IAA17, which impacts aging of *Arabidopsis*, may be part of the signaling pathway by which melatonin modulates plant aging [156].

## 8. Melatonin Improves Plant Growth

As in animals [25], melatonin has a veritable cornucopia of functions in plants [158,159]. Some of these actions are similar to those in animals, e.g., its redox functions, while others seem to be substantially different, e.g., growth promotion [64,65,72,157,158].

**Figure 9:**
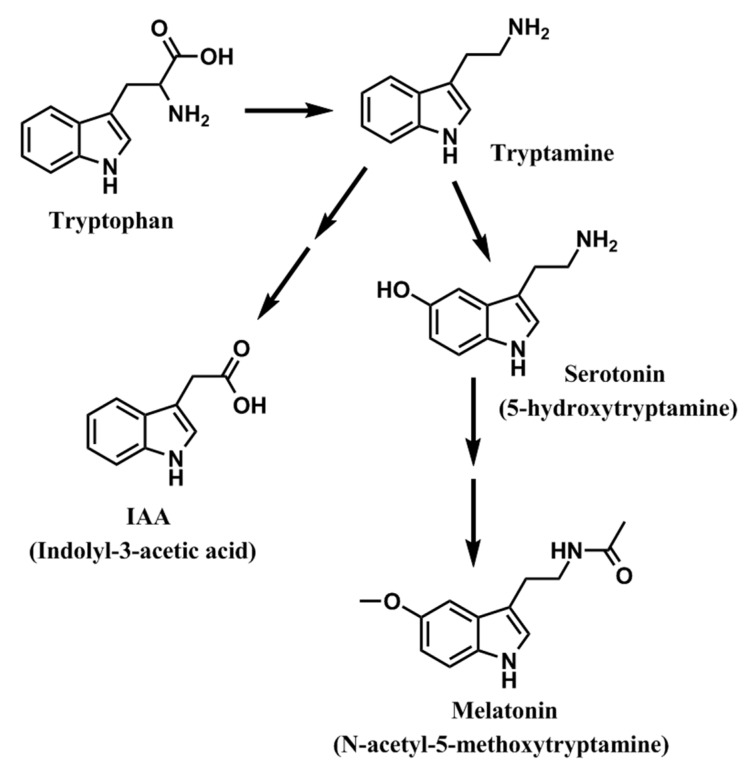
As illustrated here, tryptophan is the common precursor for the auxin indolyl-3-acetic acid (IAA) and melatonin in plants. Melatonin is reported to have auxin-like activities.

Indole compounds derived from tryptophan are common in plants. Included in this group is indolyl-3-acetic acid (IAA), a widespread auxin in plants which, among other functions, is a growth promoter [160,161]. IAA and melatonin, also a tryptophan derivative, have a markedly similar molecular structure and, since molecules with a like structure often have similar functions, melatonin was suspected of having auxin-like activity in plants. Both melatonin and IAA have an indole ring, but they vary in terms of the number of attached substituents (Figure 9). Melatonin has an acetyl group on position 3 and a methoxy group on position 5 on the indole ring while IAA has a single substituent acid group on position 3.

While melatonin enhances various parameters of growth in plants, in most cases the mechanisms are incompletely defined [158,162,163]. In this regard, melatonin has been shown to have auxin-like activities. As an example, in the etiolated lupin (*Lupinus albus*) hypocotyls melatonin at micromolar concentrations was reported to have positive growth-enhancing actions; this stimulatory effect of melatonin on growth was roughly two-thirds that of IAA [164]. Shortly after their first investigation related to melatonin’s promotion of growth in the lupin hypocotyl, this group performed a similar study using four monocot species; these included wheat (*Triticum aestirium* L.), oat (*Avena sativa* L.), barley (*Hordeum vulgare*) and canary grass (*Phalaris canariensis* L.) [165]. The actions of melatonin on growth of both the coleoptiles and roots were again compared with those of IAA. Both the coleoptiles and roots of the four monocots were assessed in terms of their longitudinal growth. The molecules were tested over a range of concentrations from 0.01 to 100 µM. While melatonin generally spurred longitudinal growth of the coleoptiles, the stimulatory actions were again less than that caused by IAA; also, melatonin’s most marked promotional effects were dose-dependent and rather specific for each plant. Also, as in their earlier report [164], the authors noted that at the highest concentration, melatonin seemingly became toxic to the plants since coleoptile growth was inhibited. The four etiolated monocot coleoptiles also took up melatonin, as measured by HPLC with electrochemical detection, with different efficiencies. The highest concentrations measured were in wheat and lowest levels were in canary grass. The measured melatonin values were poorly correlated with the growth promoting effects of the indole. Soon after the initial demonstration of the impact of melatonin on plant growth, several publications using a variety of parameters have addressed the issue of melatonin’s effects on plant growth [72,82,166,167,168]. Relative to the above-ground plant organs, the main conclusion of these studies has been that melatonin is generally beneficial to and advances their growth [162,163]. In many cases the actions of melatonin resemble those induced by the auxin, IAA.

The impact of endogenous melatonin levels on leaf growth and structure was convincingly demonstrated in a study where the concentrations of the indoleamine were lowered by generating transgenic tomato plants overexpressing rice (*Oryza sativa* L.) indoleamine dioxygenase; this enzyme metabolizes melatonin thereby keeping its levels low [169]. Characteristically, a leaf of a control wild-type tomato plant develops a terminal leaflet and two pairs of lateral leaflets in a basipetal sequence. In the plants having depressed levels of melatonin, which were measured, the leaves often had markedly different structures. In the T1 transgenic plants the number of lateral leaves was often reduced; additionally, these leaflets were malformed, being flatter than the control leaflets and their margins were less serrated. In a few cases transgenic plants developed odd-pinnately compound leaves with five or more leaflets.

Considering their results, Okazaki and co-workers [169] pointed out that IDO cleaves the indole ring of indoleamines and, therefore, the concentration of other molecules that possess a similar structure may also have been changed in the transgenic tomato plants; these perturbations may also have contributed to the abnormal leaf development. In particular, downregulation of the indoleamine, IAA, also converts compound leaves into simple leaves. The phenotypic leaf changes induced by downregulating melatonin were obviously similar to those caused by manipulation of the auxin. Hence, the precise mechanism by which the reduction in melatonin perturbed leaf development in the tomato requires additional inquiry.

Okazaki *et al.* [108] also upregulated melatonin levels in transgenic MicroTom tomato plant leaves by overexpressing the enzyme AANAT, the activity of which is typically correlated with melatonin concentrations. The leaves of some of these plants had highly increased melatonin concentrations (up to 7-fold greater than in leaves from wild type plants). Even though the leaves exhibited extremely high melatonin levels, the authors specifically mentioned no significant phenotypic changes were noted in the leaf structure. This is consistent with observations reported earlier by Murch and Saxena [170]. In this latter study, a germ plasm line of St John’s Wort (*Hypericum perforatum* L.) in which elevated melatonin levels were produced *in vitro* using mutagenized tissues exhibited structures that were morphologically similar to the wild type. Based on the outcome of the studies by Okazaki *et al.* [108] and Murch and Saxena [170], elevated melatonin levels in plants appear to be rather innocuous while depressing the levels of this indoleamine may have negative consequences on plant development.

While abnormal leaf structure is representative of morphogenetic perturbations caused by depressed melatonin levels, the functional state of these so-called deformed leaves is of great significance. At this point there are no studies of the physiology of leaves suffering with lower than normal melatonin concentrations. Conversely, with elevated levels of melatonin in plants maintain their content of β-carotenoids and increase endogenous levels of vitamin E and C and reduced glutathione [169,170]. Moreover, etiolated rice seedlings ectopically overexpressing AANAT, which augments their melatonin concentrations, also had chlorophyll levels well above those in the wild-type counterparts [171]. Considering the essential function of chlorophyll in capturing energy from photons and its role in the synthesis of critical carbohydrates, this pigment is obviously essential for health of photosynthesizing plants.

Not only is the content of chlorophyll preserved by melatonin, but its photosynthetic efficiency is likewise maintained or even enhanced. When melatonin was sprayed onto cucumber seedlings, net photosynthesis was elevated [172]. This occurred in plants maintained under room temperature conditions as well as those exposed to high ambient temperatures [173]. The judgment regarding the photosynthesis rate was based on the higher levels of leaf CO_2_, which made it available for the formation of additional carbohydrate.

Based on these limited data it seems safe to surmise that depressed melatonin levels in plants may compromise their physiology. Conversely, all indications are that higher than normal melatonin concentrations in plant organs seem to aid them in terms of thriving and surviving [174].

A well-developed root system is obviously critical for vegetative growth and seed and fruit development. An adequate root system ensures efficient water and nutrient uptake and provides a solid anchor for the plant to prevent damage resulting from movement of the above-ground tissues. The lateral roots are of special importance since they are a highly dynamic and physiologically active component of the root system. Also of importance is that root architecture is plastic and exhibits marked changes depending on the nutrient content of the soil, soil matrix heterogeneity and biotic interactions [175]. Lateral root growth is a highly complex process and is regulated to a large degree by auxin [176,177].

Recently, reports have surfaced which reveal that melatonin also exercises some control over root architecture as observed in St. John’s Wort, wild leaf mustard, sweet cherry root stocks and lupin [178,179,180,181]. In each of these studies the ability of melatonin to enhance lateral root growth duplicated the actions of IAA. Another feature that became apparent in two of these reports [179,180] is that at least for lateral root elongation the response to melatonin may be concentration specific. Thus, lower levels of melatonin were more effective inducers of root branching than were the higher doses that were tested; indeed, at the upper extreme of concentrations of melatonin (10–100 µM) used, the indole may stymie lateral root growth.

While the actions of melatonin on rooting have been described as being auxin-like, the data obtained by Pelagio-Flores *et al.* [182] indicate that this function is independent of IAA. This was documented using auxin-responsive marker constructs in the seedlings of *Arabidopsis thaliana*. The outcome of these studies was that melatonin did not activate the expression of auxin-inducible genes which drive morphogenetic root growth. In contrast to other reports [179,180], these workers also did not find that high concentrations of melatonin (up to 600 µM) interfered with root branching in *Arabidopsis*, a species not used in the other two publications.

**Figure 10:**
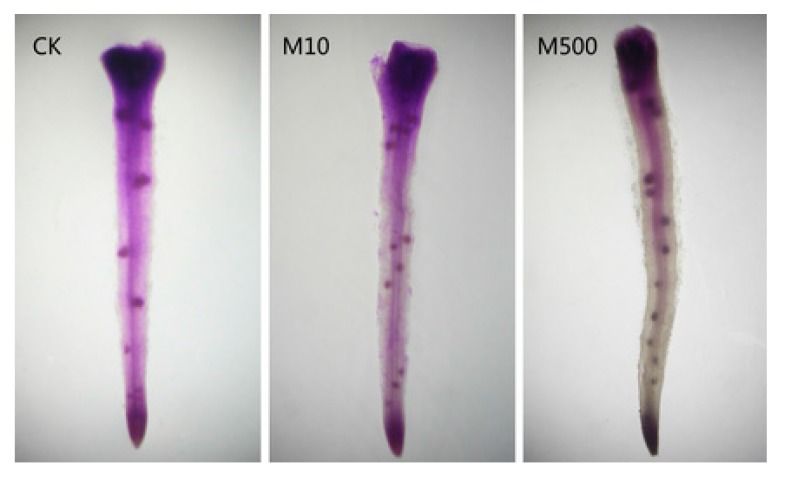
Feulgen stained cucumber roots reveal lateral root primordia (dark points). Melatonin at concentrations of either 10 µM (M10) or 500 µM (M500) stimulated lateral root primordia over the number seen in control roots (CK). From Zhang *et al.* [183] with permission.

A serious attempt to define the genes regulated by melatonin which determine lateral root growth was carried out by Zhang and colleagues [183]. The seeds of the cucurbitaceous plant, the cucumber, were used given that the genomic sequence of this species is known. The authors used RNA sequencing to explore the potential mechanisms of induction of lateral root growth that was observed in other reports [178,179,180,181]. The seeds were primed in a solution containing either 10 µM or 500 µM melatonin and germinated in a 100 mM NaCl solution for 48 h. In seeds primed with a 500 µM melatonin solution, 121 genes were significantly upregulated in the seedlings while 196 genes were downregulated. On the basis of their expression parameters, the peroxidase-related genes where those likely related to melatonin’s stimulatory action on lateral root growth. However, genes related to cell wall formation, carbohydrate metabolism, oxidation/reduction processes and catalytic activity also exhibited changes in gene expression patterns; these multiple, diverse changes precluded the identification of a definitive process(es) by which melatonin mediates its effect on lateral root elongation. Some roots were also Feulgen stained [184] to identify lateral root primordia. This showed that melatonin clearly augmented the numbers of primordial root sites supporting its stimulatory action on lateral root growth [183] (Figure 10).

Transgenic rice plants (*Oryza sativa* cv. Dongjin) rich in melatonin due to over expression of sheep SNAT exhibited greater biomass but delayed flowering and crop yield [184,185]. This indicates that high constitutively-expressed melatonin levels may yield different results in terms of product yield than when plants are germinated from seeds that are primed with a melatonin solution [186]. As will be summarized below, priming of seeds in a solution of melatonin has been shown to enhance germination, improve plant height and biomass and augment crop yield.

## 9. Melatonin Improves Crop Yield and Provides Crop Protection

The most thorough and potentially important investigation related to the ability of melatonin to impact crop yield is that of Wei and co-workers [187] who used the soybean as the model plant. Soybean is one of the most valuable agricultural crops in the world; it is widely used for seed oil production, as feed for livestock, for biofuel feedstock and as an important source of protein in the human diet [188]. The tremendous value of this crop is emphasized by the fact that the world production has increased steadily since 1961 and is forecast to reach 372 million tons by the year 2030. To achieve this goal, the soybean yield from available cultivated land must be increased substantially since land used for this purpose is predicted to remain stable or may actually decrease. Because of this, there is an urgent and critical need to identify means to augment soybean production on the current field allotment [189]. Obviously, any means that would contribute to achieving this goal of enlarging soybean production, or of any agriproduct (see below), would be of major economic importance.

As one aspect of a study designed to examine the action of melatonin on growth and abiotic stress tolerance of soybean plants grown from melatonin-treated seeds, Wei *et al.* [187] also measured the yield of soybeans. For the experiment, soybeans (*Glycine max SuiNong 28 SN28*) were initially coated with 500 µL/100 seed reagent that contained no melatonin or melatonin at concentrations of either 50 µM or 100 µM. After coating, the seeds were dried at room temperature and then sowed in pre-watered soil. After germination, the seedlings were grown in a sunlit greenhouse located at 40°22'N and 116°22'E (Beijing, China). The agronomic traits that were recorded included the number of soybean pods per plant, the number of seeds per pod and 100-seed weight.

The melatonin concentrations selected for this study were based on observations of Hernandez-Ruiz *et al.* [164] which suggested that 200 µM concentrations of melatonin improved the growth of lupin plants. Wei *et al.* [187] found that the 50 µM and 500 µM melatonin exposure improved seed germination and the plants developed larger leaves; this was statistically verified when the trifoliate leaves of 5-week-old melatonin-treated plants were compared with those from control plants. At 3 months after germination, the agroeconomic measures documented that the soybean plants grown from melatonin-coated seeds had more pods per plant and more seeds per pod. The 100-seed weight did not differ between the melatonin-treated and control plants. All other parameters examined in this study also indicated that melatonin-treated plants are hardier than their control counterparts.

A similar stimulatory effect of melatonin on corn and cucumber production was noted by Posmyk and colleagues [140,159,174]. In these studies, rather than coating the seeds with melatonin as described by Wei *et al.* [187], the authors primed the seeds overnight in a melatonin solution. The priming caused a marked increase in the melatonin levels in the seeds and, when they were germinated and grown to maturity, the resulting plants bore more product, *i.e.*, corn and cucumber, than did the plants grown from seeds primed only in water.

It is critically important that studies such as those of Wei *et al.* [187] and Posmyk and colleagues [140,159] be expanded. If improved crop yield is verified in large scale field trials, melatonin could prove to be, at least in part, a solution to the problem of producing more product without increased land usage. Melatonin is easy to synthesize in pure form and it is inexpensive, so its use could prove to be a practical application of this indoleamine.

The results summarized above could also be of special interest for another reason. The reports show that the application of exogenous melatonin to seeds, either by coating or priming, had an impact on plant growth and crop production throughout the life cycle of the plant [140,159,174,187]. Since it would seemingly not be difficult on a large scale basis to pre-treat seeds with melatonin prior to sowing as described, the application of melatonin to improve agricultural production would likely be doable. It would also be of interest to determine whether plants germinated and grown from melatonin-treated seeds are more stress tolerant or whether the nutrient composition of the crop was changed.

**Figure 11:**
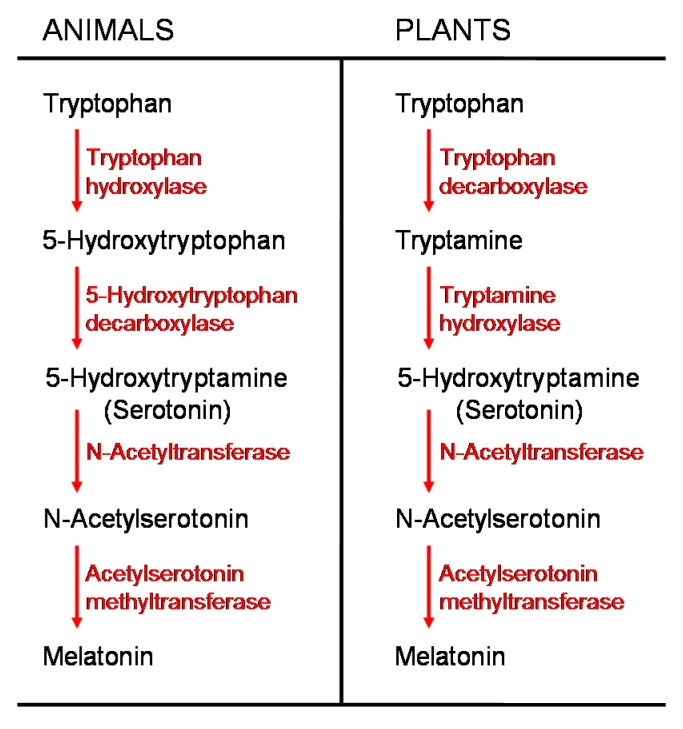
Pathways of melatonin synthesis from tryptophan in animals and plants. The first two steps in the pathway, *i.e.*, hydroxylation and decarboxylation, are reversed in plants relative to animals. The last two steps are the same.

Considering the described importance of exogenously-applied melatonin to enriching crop yield, the consequences of upregulating endogenous melatonin synthesis in plants should be assessed in terms of crop production. Within the last several years remarkable advances have been made and new information has been uncovered related to the pathway of melatonin biosynthesis in plants [190,191,192,193]. While this route differs slightly from that in animals [150,194,195] (Figure 11), nevertheless, tryptophan is the common precursor in all species and melatonin formation from serotonin is the same two-step process in plants as in animals [191,192,196]. In plants, chloroplasts may be a major site of melatonin production [197,198].

**Figure 12:**
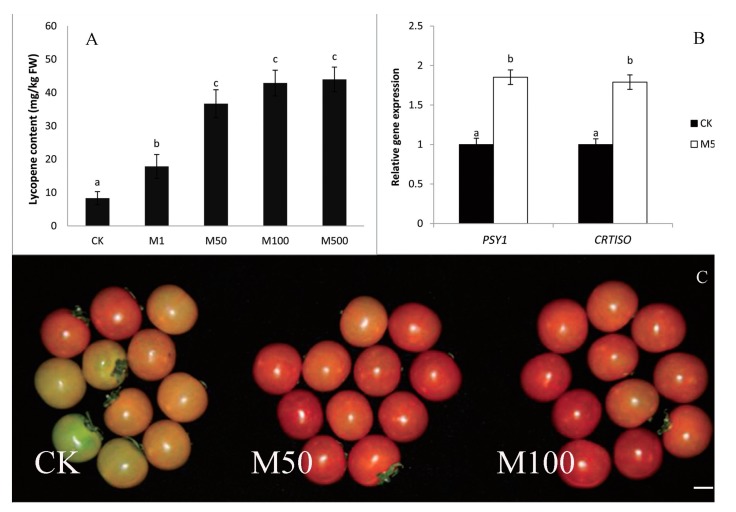
Influence of melatonin pigment accumulation in Bmei cherry tomatoes. Freshly-picked green tomatoes were insulated in a solution containing 1 µM (M1), 50 µM (M50), 100 µM (M100) or 500 µM (M500) melatonin solution for 45 minutes. Thereafter, the tomatoes were stored for 25 days. Melatonin hastens lycopene (**A**) accumulation in a concentration-dependent manner; (**B**) PCR analysis of PSY1 and CRTISO gene expression in control (CK) and 50 µM-treated tomatoes; PSY1 and CRTISO are involved in lycopene synthesis; (**C**) Tomatoes treated without melatonin (CK) or with different concentrations of melatonin (M50 or M100); see text for details. Bars with different letters (a,b or c) differ statistically significantly. From Sun *et al.* [202] with permission.

The genes for the plant melatonin-synthetic enzymes have also been cloned [199,200]. The manipulation of endogenous melatonin synthesis in plants using transgenic technologies is certainly feasible and has already been done for two species [129,201]. Genetic modulation of the endogenous melatonin pathway in plants for the purpose of enhancing crop production, however, has not been accomplished.Sun and colleagues [202] tested melatonin’s effects on post-harvest ripening of Bmei cherry tomatoes. The fruits were collected at their green stage of development. After harvesting, they were placed in one of several solutions of melatonin (1, 50, 100 or 500 µM) for 2 h. Thereafter, the tomatoes were kept at a temperature of 15 °C and 80% relative humidity for 25 days. Melatonin exposure markedly advanced lycopene levels and color development (Figure 12) as well as stimulating the expression of several key genes including those of phytoene synthase 1, carotenoid isomerase and aquaporins.

Additionally, melatonin-treated fruits exhibited significantly accelerated fruit softening, elevated water soluble pectin and diminished protopectin. These changes were accompanied by upregulation of cell wall modifying proteins including polygalacturonase, pectin isomerase 1, β-galactosidase and expansion 1. Melatonin also influenced ethylene synthesis, ethylene perception and ethylene signaling. Ethylene plays a major role during ripening of tomatoes due to is regulatory actions or carotenoid lycopene synthesis, enhancing degradation of the cell wall and converting starch to sugars [203]. It is clear from these findings that melatonin hastened the development of color and flavor of the tomato fruit via its action on ethylene synthesis in the post-harvest state. Color and flavor are, of course, major discernible aspects of fruit quality. These findings are applicable not only to tomato but likely to other horticultural products as well. Improving product quality reduces product wastage.

## 10. Plant Melatonin: Rules of Engagement

In animals, melatonin has an uncanny diversity of means by which it can impact cellular organelles and organismal physiology. In vertebrates, membrane melatonin receptors, functioning under the monikers MT1 and MT2, have been identified, characterized and cloned [204,205,206]. These receptors are extremely widespread in animals and are accepted as mediating many of the actions of melatonin [207,208,209,210,211]. Nuclear receptors (binding sites), although of questionable existence and certainly less well defined than the membrane molecules, have also been proposed and may be operative in animal cells [212,213,214]. Additionally, there are cytosolic locations where melatonin may link up with other targets [215,216,217,218,219] that result in downstream alterations in cellular physiology. To date, none of the receptors (binding sites) referred to above have been identified in plant cells. This may not be because they do not exist but, rather, related to the fact that no one has looked for them.

What is clearly obvious is that, as in animal tissues, melatonin reduces oxidative stress in plants. In vertebrates, this protection is a consequence of the direct scavenging actions of melatonin [38,55,220,221,222,223] and its metabolic kin [56,57,58,60,61,62]. The antioxidant actions of melatonin in plants very likely stems from some of these same actions. In addition to the direct detoxification of reactive oxygen and reactive nitrogen species in animals, melatonin also promotes the activities of antioxidative enzymes [111,224] and glutathione production [25,113], a potent antioxidant in its own right. These functions of melatonin are believed to involve its interaction with receptors [114,218]. Since melatonin has similar functions in plants [173], it portends the existence of some type of receptor or binding molecule in these species. It is anticipated these issues will be resolved as research on melatonin’s actions in plants continues to evolve.

## 11. Concluding Remarks

Melatonin is a remarkably, heterogeneously-functioning beneficial molecule in plants as in animals. Its discovery in land plants two decades ago [32,39] has led to a burgeoning investigative field that has already made substantial advances in uncovering the marked actions of this versatile indoleamine. The presence of melatonin in plants has implications not only for plant growth and crop yield but also in terms of animal and human nutrition. When melatonin-containing plant products are consumed, the indoleamine is absorbed after which it performs its functions at the cellular levels. In animals, as in plants, melatonin is a highly metabolically useful molecule which neutralizes pathophysiological processes that compromise healthy living [25,45,59].

Melatonin appears to be no less important in plants. And its actions likely contribute to the ecological success of plants and their ability to produce agriproducts. As summarized herein, melatonin aids seeds in germinating, improves plant development and maturation of both the root system and above ground tissues [132,136,138,144], protects plants from abiotic [110,124,141,145] and biotic stresses [149] which, because they are sessile, cannot be avoided. In doing so plants, because they contain melatonin, exhibit an increased tolerance to environmental insults and can, in fact, acutely improve their defensive posture by upregulating endogenous melatonin production [159,162]. One fallout of the improved performance of plants because they possess melatonin is augmented seed production. Greater seed availability increases the likelihood of their more widespread dissemination which contributes to the growth of its species in more habitats. Finally, healthier plants have the capability of producing a rich yield of edible products which obviously benefits the species that consume vegetation for nutrients.

Beyond this, the potential utility of melatonin-enriched plants as to their phytoremediative capacity has yet to be exploited [83,121]. Moreover, melatonin may be found to be useful for the protection or preservation of endangered plant species [225]. Of the numerous gaps in the knowledge regarding the actions of melatonin in plants, a major one relates to the specific mechanisms by which the indole mediates its actions. In animals, melatonin has both receptor-mediated and receptor-independent actions [25,26]. The best known melatonin receptors (MT1 and MT2) are located on the membranes of cells and many of the signal transduction mechanisms have been defined [204,205,206,207,208,209,210,211]. Yet, no group has examined plant cells for the presence of similar receptor proteins. Some intracellular actions of melatonin in animal cells also may involve its binding to cytosolic or nuclear molecules [212,213,214,215,216,217]; again, these potential sites have gone uninvestigated in plant cells.

Non-receptor-mediate actions of melatonin in animal cells are linked to the ability of melatonin and its metabolites to function as free radical scavengers and antioxidants [25,26,38,56,57,58,59,60,61,62,88]. To date, a majority of the functions of melatonin in plant cells have likewise been attributed to the antioxidant effects of the indole [102,105,110,115,117,121,130]. Clearly, the mechanisms whereby melatonin carries out its multiple functions in plant cells still requires extensive investigative effort. It seems obvious from the data summarized herein that melatonin is a highly useful molecule which contributes significantly to the hardiness of plants. In doing so, it aids plants to not only survive but, more importantly, to thrive.
